# Supplementary material for: Observation of quantum oscillations near the Mott-Ioffe-Regel limit in CaAs3
Source: Natl Sci Rev. 2024 Mar 29;11(12):nwae127. doi: 10.1093/nsr/nwae127 (PMC11660949; doi:10.1093/nsr/nwae127)
Supplement: nwae127_Supplemental_File [file nwae127_supplemental_file.docx]

**Supplementary Notes for**

**Observation of quantum oscillations near the Mott-Ioffe-Regel limit in CaAs_3_**

Yuxiang Wang^1#^, Minhao Zhao^2#^, Jinglei Zhang^4#^, Wenbin Wu^5^, Shichao Li^6^, Yong Zhang^4^, Wenxiang Jiang^7^, Nesta Benno Joseph^8^, Liangcai Xu^9^, Yicheng Mou^1^, Yunkun Yang^2^, Pengliang Leng^2^, Yong Zhang^10^, Li Pi^4^, Alexey Suslov^11^, Mykhaylo Ozerov^11^, Jan Wyzula^12^, Milan Orlita^12^, Fengfeng Zhu^7^, Yi Zhang^13^, Xufeng Kou^10^, Zengwei Zhu^9^, Awadhesh Narayan^8^, Dong Qian^7^, Jinsheng Wen^6^, Xiang Yuan^5,14*^, Faxian Xiu^1,2,3,15,16*^, Cheng Zhang^1,15*^

^1^ State Key Laboratory of Surface Physics and Institute for Nanoelectronic Devices and Quantum Computing, Fudan University, Shanghai 200433, China

^2^ State Key Laboratory of Surface Physics and Department of Physics, Fudan University, Shanghai 200433, China

^3^ Shanghai Qi Zhi Institute, 41st Floor, AI Tower, No. 701 Yunjin Road, Xuhui District, Shanghai, 200232, China

^4^ Anhui Province Key Laboratory of Condensed Matter Physics at Extreme Conditions, High Magnetic Field Laboratory of the Chinese Academy of Sciences, Hefei 230031, China

^5^ State Key Laboratory of Precision Spectroscopy, East China Normal University, Shanghai 200241, China

^6^ National Laboratory of Solid State Microstructures and Department of Physics, Nanjing University, Nanjing 210093, China

^7^ Key Laboratory of Artificial Structures and Quantum Control (Ministry of Education), School of Physics and Astronomy, Shanghai Jiao Tong University, Shanghai 200240, China

^8^ Solid State and Structural Chemistry Unit, Indian Institute of Science, Bangalore 560012, India

^9^ Wuhan National High Magnetic Field Center and School of Physics, Huazhong University of Science and Technology, Wuhan 430074, China

^10^ School of Information Science and Technology, ShanghaiTech University, Shanghai 201210, China

^11^ National High Magnetic Field Laboratory, Tallahassee, Florida 32310, USA

^12^ Laboratoire National des Champs Magnétiques Intenses, CNRS-UGA-UPS-INSA-EMFL, 25, rue des Martyrs, 38042 Grenoble, France

^13^ International Center for Quantum Materials, School of Physics, Peking University, Beijing 100871, China

^14^ School of Physics and Electronic Science, East China Normal University, Shanghai 200241, China

^15^ Zhangjiang Fudan International Innovation Center, Fudan University, Shanghai 201210, China

^16^ Shanghai Research Center for Quantum Sciences, Shanghai 201315, China

^#^ These authors contributed equally to this work

**^*^** Correspondence and requests for materials should be addressed to X. Y. (E-mail: [xyuan@lps.ecnu.edu.cn](mailto:xyuan@lps.ecnu.edu.cn)), F. X. (E-mail: [Faxian@fudan.edu.cn](mailto:Faxian@fudan.edu.cn)) & C. Z. (E-mail: [zhangcheng@fudan.edu.cn](mailto:Zhangcheng@fudan.edu.cn))

**Supplementary Note 1: Low-energy Hamiltonian and optical selection rules of CaAs_3_.**

Based on the ARPES result, the bulk band of CaAs_3_ can be described by a standard massive Dirac Hamiltonian as,

$$\begin{aligned} H\left( \mathbf{k} \right)=\Delta\tau_{z}\sigma_{0}+\hbar v_{Fx}k_{x}\tau_{x}\sigma_{z}+\hbar v_{Fy}k_{y}\tau_{y}\sigma_{0}+\hbar v_{Fz}k_{z}\tau_{x}\sigma_{x},\#\left( 1 \right) \end{aligned}$$

where $\mathbf{k}$ is the momentum; $\hbar$ is the reduced Planck’s constant; and $\sigma, \tau$ are the Pauli matrices for the spin and orbital degree of freedom, respectively. The band structure is determined by the parameters $v_{F}$ and $\Delta\equiv E_{g}/2$, which are known as Fermi velocity and mass term, respectively.

**i. Landau quantization with magnetic field applied on the z-axis**

Landau level spectrum can be further obtained by performing Peierls substitution in Eq. (1) which transforms $\mathbf{k}$ to $\boldsymbol{\pi=k+}e\boldsymbol{A/\hbar}$, with the vector potential $\mathbf{A}$ determined by magnetic field $\mathbf{B=}\nabla\boldsymbol{\times A}$. Introducing the parabolic dispersion approximation near the vanishing momentum (only valid at the band edge), density of states of the formed 1D Landau bands is found to be divergent at the $k_{z}=0$, which dominates the Landau level spectroscopy. At the zero-momentum point, the Hamiltonian is rewritten as two decoupled two-band Hamiltonians,

$$H_{0}=\left( \begin{matrix} h_{1} & 0 \\ 0 & h_{2} \end{matrix} \right),$$

$$\begin{aligned} h_{1}=\left( \begin{matrix} \Delta& \sqrt{2eB\hbar}v_{F}a \\ \sqrt{2eB\hbar}v_{F}a^{\dagger} & -\Delta\end{matrix} \right),h_{2}=\left( \begin{matrix} \Delta& -\sqrt{2eB\hbar}v_{F}a^{\dagger} \\ -\sqrt{2eB\hbar}v_{F}a & -\Delta\end{matrix} \right),\#\left( 2 \right) \end{aligned}$$

where $v_{F}=\sqrt{v_{Fx}v_{Fy}}$ is the geometric average of the Fermi velocity along the in-plane directions, and $e$ is the elementary charge. Two ladder operators are defined as,

$$\begin{aligned} a=\sqrt{\frac{\hbar}{2eB}} \left( \pi_{x}-i\pi_{y} \right), a^{\dagger}=\sqrt{\frac{\hbar}{2eB}}\left( \pi_{x}+i\pi_{y} \right).\#\left( 3 \right) \end{aligned}$$

Introducing eigenvector basis $\phi_{n}=(|n-1\rangle,|n\rangle,|n\rangle,|n-1\rangle)^{T}$ where $a^{\dagger}a\left| n \right\rangle=n\left| n \right\rangle$, sub-Hamiltonian $h_{1}$ reads,

$$\begin{aligned} h_{1}=\left( \begin{matrix} \Delta& \sqrt{2eB\hbar v_{F}^{2}n} \\ \sqrt{2eB\hbar v_{F}^{2}n} & -\Delta\end{matrix} \right).\#\left( 4 \right) \end{aligned}$$

The corresponding Landau level spectrum is directly obtained,

$$\begin{aligned} E_{n}=\pm\sqrt{\Delta^{2}+2eB\hbar v_{F}^{2}n}.\#\left( 5 \right) \end{aligned}$$

By the same method, the Landau level of sub-Hamiltonian $h_{2}$ can be derived, which is identical to Eq. (5).

**ii. Optical selection rules**

The optical selection rule is determined by the transition matrix element $\langle\phi_{n^{'}}\left| v^{\pm} \right|\phi_{n}\rangle$, where velocity operators $v^{\pm}=\frac{\sqrt{2}}{2}\left( v_{x}\pm iv_{y} \right)$ with $v_{i}=\frac{1}{\hbar}\frac{\partial H_{0}}{\partial\pi_{i}}, i=x,y$correspond to the right-handed (+) and left-handed ($-$) circular polarized light, and $\left| \phi_{n^{'}} \right\rangle, |\phi_{n}\rangle$ are the eigenstates denoting the final and initial state, respectively. The velocity operators in matrix form read,

$$\begin{aligned} v^{+}=\sqrt{2}\left( \begin{matrix} 0 & v_{F} & 0 & 0 \\ 0 & 0 & 0 & 0 \\ 0 & 0 & 0 & 0 \\ 0 & 0 & -v_{F} & 0 \end{matrix} \right),v^{-}=\sqrt{2}\left( \begin{matrix} 0 & 0 & 0 & 0 \\ v_{F} & 0 & 0 & 0 \\ 0 & 0 & 0 & -v_{F} \\ 0 & 0 & 0 & 0 \end{matrix} \right).\#\left( 6 \right) \end{aligned}$$

According to the calculated transition matrix $\langle\phi_{n^{'}}\left| v^{\pm} \right|\phi_{n}\rangle\propto\delta_{{|n}^{'}|,|n|\pm1}$, the optical selection rules of interband-Landau-level transitions are summarized as $\Delta\left| n \right|=+1$ and $\Delta\left| n \right|=-1$ for right-handed and left-handed circular polarized light, respectively. Due to the identical transition energy of $-n\to n+1$ and $-\left( n+1 \right)\to n$, these two series of Landau level resonance can’t be distinguished in nonpolarized magneto-infrared spectroscopy. As for the intraband-Landau-level transitions $n\to n+1$, they are also allowed when the Fermi level is located at the conduction band. However, in our experiment, these transitions are not observed since the corresponding energy is far below the experimental spectral (mid-infrared) range. The intraband-Landau-level transitions are expected to approach zero energy when the external magnetic field vanishes.

**iii. Dirac mass extracted from magneto-infrared spectroscopy**

The in-plane energy dispersion of CaAs_3_ $E(k)=\pm\sqrt{{{(E}_{g}/2)}^{2}+\left( \hbar v_{F}k \right)^{2}}$can be deduced by the band parameters fitted from interband-Landau-level transitions with described massive Dirac model

$$\begin{aligned} \omega_{n}\left( B \right)=\sqrt{2{e\hbar Bv}_{F}^{2}\left( \left| n \right|+1 \right)+\Delta^{2}}+\sqrt{2{e\hbar Bv}_{F}^{2}\left| n \right|+\Delta^{2}}.\#\left( 7 \right) \end{aligned}$$

where $n$ is the Landau index; the value of $\Delta$ equals half of the energy gap $E_{g}$. The quasi-particle mass can be derived by the fitting parameter $m_{D}=\frac{E_{g}}{{2v}_{F}^{2}}$ which is also known as Dirac mass. In our experimental regime, CaAs_3_ fully reaches quantum limit (zero Fermi energy, high magnetic field). The mass extracted by the magneto-infrared spectrum is mainly determined by the band parameter and hardly influenced by the position of the Fermi level. However, transport measurement is sensitive to the electronic state near the Fermi level with extracted mass more susceptible to the Fermi energy and interaction effect.

We also briefly discuss the difference of $m^{*}$ given by magneto-infrared spectroscopy and quantum oscillation. For the classical system described by the Schrödinger equation, $m^{*}$can be directly extracted from the cyclotron frequency $\omega_{c}=eB/m^{*}$measured in magneto-infrared spectroscopy. However, for the massless Dirac system, the effective mass is not well-defined. The cyclotron mass can only be measured in the classical limit (high Fermi energy, low magnetic field), which is proportional to Fermi energy.^1,2^ The energy of Landau level formed by massless Dirac fermion follows $\sqrt{2e\hbar Bv_{F}^{2}\left| n \right|}$, which is only determined by the Fermi velocity. Therefore, for the massive Dirac system CaAs_3_, the mass extracted from the magneto-infrared spectroscopy serves as single particle mass that is directly determined by the non-interacting band parameter, but the mass obtained from the magneto-transport is influenced by the Fermi surface property.

**Supplementary Note 2: Excluding the surface state as the origin of quantum oscillations.**

Since CaAs_3_ has been proposed as a candidate for node line semimetals^3,4^, it is important to clarify whether the quantum oscillations are from the topological surface states or other type of surface state due to band bending. We carefully exclude the surface origin of quantum oscillations based on ARPES, Hall effect, Shubnikov–de Haas (SdH) oscillations, and magneto-infrared spectrum as follows:

(1) Three-dimensional (3D) Fermi surface: Most important evidence is given by the quantum oscillations in CaAs_3_ which evolve continually with the rotation of magnetic fields, corresponding to a 3D Fermi surface instead of a two-dimensional (2D) one. The effective mass value from quantum oscillations (Fig. 2g) is much larger than that of typical topological surface states ($m^{*}\ll m_{e}$)^5–8^. Meanwhile, the quantum oscillations show similar frequency among different samples and are robust enough to survive after four years in air (Fig. S10). These features are clearly different from surface-related quantum oscillations^5–8^.

(2) Bulk nature of measured electronic band: The node line semimetal phase of CaAs_3_ is calculated in the absence of spin-orbit coupling. In real materials, the effect of spin-orbit coupling will open up a bulk band gap and transform the system into either a normal insulator or a topological insulator. According to previous study^9^ and our work, no surface state is detected in the bulk gap, suggesting a normal insulator phase in CaAs_3_. Photon-energy-dependent measurements were also carried out to verify the bulk nature of the observed bands^9^. The magneto-infrared spectrum points to a gapped Dirac model for the Landau-quantized band, which have the similar gap value and dispersion as the bulk band determined by ARPES. And the presence of low-index interband-Landau-level transition agrees with the ARPES result in Fig. 1c, where the Fermi level stays just above the edge of bulk conduction band.

(3) Exclusion of 2D carrier density: By assuming the SdH oscillations originating from a 2D surface layer, the surface carrier density can then be extracted as $n_{s}=\frac{k_{F}^{2}}{2\pi}=$1.27×10^12^ cm^-2^ from the Fermi wave vector. Divided by the total thickness of the crystal, the derived 3D density is around 4.24×10^13^ cm^-3^, two orders of magnitude smaller than the Hall density at 2.5 K. It then suggests that the Hall effect is dominated by the bulk channel, which is inconsistent with the large oscillation amplitude in Hall effect shown in Fig. 2a and Fig. S1e.

Therefore, we can safely exclude the surface state as the origin of quantum oscillations in CaAs_3_.

**Supplementary Note 3: Discussion of spatial** **inhomogeneity.**

In the above section, we have excluded the possibility of surface-related quantum oscillation. Since only SdH oscillations are detected without de Haas–van Alphen (dHvA) oscillations, one needs to carefully address whether prominent spatial inhomogeneity is presented in the sample so that the oscillation regions are comparably small in volume (Fig. S12). To exclude possible inhomogeneity in chemical composition or crystalline lattice, we measure XRD and energy dispersive spectroscopy (EDS) of the CaAs_3_ crystals as shown in Fig. S1 a and f. These results show uniform chemical stoichiometry and good crystalline property with (010) surface in the crystal. No other element is introduced as flux or doping during the growth. More importantly, both ARPES and magneto-infrared spectroscopy yield similar band structure with consistent Fermi wave vector value given by quantum oscillations. Note that these two spectroscopic approaches collect signals from the crystal macroscopically. And typical penetration depth for infrared spectroscopy here is in the micrometer scale. Hence, they suggest that the Landau quantized band dominates the electronic structure in the crystal rather than only occupying a small volume as in Fig. S12.

**Supplementary Note 4: Difference with other insulating systems showing quantum oscillations.**

SmB_6_, YbB_12_ and monolayer WTe_2_ are gapped charge insulators without electronic bulk Fermi surfaces due to many-body effects^10–14^. Therefore, the quantum oscillations in these three systems are considered to originating from charge-neutral quasiparticles rather than electrons. Distinct from these systems, CaAs_3_ has an electronic Fermi surface and the insulating-like temperature dependence of resistivity. The carrier density extracted from the volume of Fermi sphere is almost three orders of magnitude larger than that extracted from Hall coefficient, suggesting most of the electrons were localized. The anomalous quantum oscillations in CaAs_3_ go beyond the traditional picture of hopping conduction near Mott-Ioffe-Regel limit.

**Supplementary Note 5: Effective mass fitted by different methods.**

In order to further improve the reliability of the effective mass calculation, we try two additional methods to extract the effective mass *m*^*^. The first one is by calculating *m^*^* from the second derivative of MR with no need for background subtraction. We start from the Lifshitz-Kosevich (LK) formula $\frac{{\Delta\rho}_{xx}}{\rho_{BKG}}R_{T}R_{D}cos[2\pi(\frac{F}{B}+\varphi)]$ and perform the first derivative, which gives:

$$\begin{aligned} \frac{\partial\frac{{\Delta\rho}_{xx}}{\rho_{BKG}}}{\partial B}{\frac{2\pi F}{B^{2}}R}_{T}R_{D}\sin\left[ 2\pi\left( \frac{F}{B}+\varphi\right) \right]+\frac{\partial R_{T}}{\partial B}R_{D}\cos\left[ 2\pi\left( \frac{F}{B}+\varphi\right) \right]+R_{T}\frac{\partial R_{D}}{\partial B}\cos\left[ 2\pi\left( \frac{F}{B}+\varphi\right) \right]\#\left( 8 \right) \end{aligned}$$

where ${\Delta\rho}_{xx}=\rho_{xx}-\rho_{BKG}$ and the left part could be simplified to $\frac{\partial\rho_{xx}}{\partial B}$ since $\frac{\partial{\Delta\rho}_{xx}}{\partial B}$ is much larger than $\frac{\partial\rho_{BKG}}{\partial B}$. By ignoring the field dependence of $R_{T}$ and $R_{D}$, the first derivative of LK formula could be simplified to $\frac{\partial\rho_{xx}}{\partial B}{\frac{1}{B^{2}}R}_{T}R_{D}sin[2\pi(\frac{F}{B}+\varphi)]$. Hence the second derivative of LK formula can be simplified to:

$$\begin{aligned} \frac{\partial^{2}\rho_{xx}}{\partial B^{2}} -{\frac{1}{B^{4}}R}_{T}R_{D}\cos\left[ 2\pi\left( \frac{F}{B}+\varphi\right) \right]\#\left( 9 \right) \end{aligned}$$

In Fig. S13a, we plotted the comparison of second derivative of MR and extracted oscillations. The π phase shift between these two curves verifies the validity of this simplified second derivative formula. We then calculate *m^*^* based on the temperature dependence of $\frac{\partial^{2}\rho_{xx}}{\partial B^{2}}$ through $B^{4}\frac{\partial^{2}\rho_{xx}}{\partial B^{2}} R_{T}R_{D}cos[2\pi(\frac{F}{B}+\varphi)]$. Fig. S13b plots the obtained $B^{4}\frac{\partial^{2}\rho_{xx}}{\partial B^{2}}$ at different temperatures. The fitted *m^*^* of four oscillation peaks at different magnetic fields is shown in Fig. S13c and summarized as a red dotted curve in Fig. S13d. It is clear that *m^*^* calculated by the second derivative method presents consistent field dependence.

In addition to the second derivative method presented above, we also use another area-integral method to verify the quasiparticle mass enhancement. Through a similar simplification process of the second derivative method, the integral of the LK formula is approximated as:

$$\begin{aligned} \int\frac{{\Delta\rho}_{xx}}{\rho_{BKG}}dB\propto-{B^{2}R}_{T}R_{D}\sin\left[ 2\pi\left( \frac{F}{B}+\varphi\right) \right]\#\left( 10 \right) \end{aligned}$$

We now consider a narrow integral area $S_{osc}=\int_{\frac{1}{\frac{1}{B_{0}}+\frac{1}{10F}}}^{\frac{1}{B_{0}}-\frac{1}{10F}} {\Delta\rho}_{xx}dB$ around the oscillation peak/valley positions at MR curve, where $B_{0}$ is the oscillation peak/valley positions determined by the Landau fan diagram. Here *F* is the frequency of oscillations. In Fig. S14a-b, we show how the integral region is obtained from the MR curve by subtracting the baseline at different temperatures. We then use the area of shadow regions $S_{osc}$ at different temperatures in Fig. S14b to calculate *m^*^* based on ${\frac{S_{osc}}{{B_{0}}^{2}}\propto R}_{T}R_{D}sin[2\pi(\frac{F}{B}+\varphi)]$. Since the extracted regions now only occupy 20% of the whole period where the oscillations are most pronounced, it can largely eliminate the influence of possible improper MR background subtraction. The fitted *m^*^* value of four oscillation peaks is shown in Fig. S14c and summarized as a blue dotted curve in Fig. S14d. It shows similar effective mass value and field dependence as the one from the background subtraction method.

The three methods presented above yields similar effective mass value and consistent field dependence as shown in Fig .S14e, especially considering the latter two does not directly involve the fitting of total MR ground over a large range.

**Supplementary Note 6: Simulation of conductivity and magnetic susceptibility**

In the view of magnetization, the localized electrons contribute a noncoherent magnetization signal, which overwhelms the de Haas–van Alphen (dHvA) oscillation. However, for magneto transport, these noncoherent signal in conductivity is excluded due to the Anderson localization, which makes the SdH oscillations easier to be detected. To verify this statement, we made a simulation of conductivity and magnetic susceptibility without the mobility edge. If there is no mobility edge (Fig. S16d), these localized electrons will be mobile and contribute a background conductivity $\sigma_{xx}^{BG}\left（ B=0 \right）=\left( \frac{n_{SdH}}{n_{H}}-1 \right)*\sigma_{xx}\left（ B=0 \right）$, where $\sigma_{xx}$ is measured conductivity in experiment (yellow line in Fig. S16(a)). Assume the $\sigma_{xx}^{BG}$ obey a quadratic magnetoresistance $\sigma_{xx}^{BG}(B)= \sigma_{xx}^{BG}\left（ B=0 \right）/(1+{(\mu*B)}^{2})$, then we can get the simulated conductivity without the mobility edge $\sigma_{xx}^{Simu}(B)=\sigma_{xx}^{BG}(B)+\sigma_{xx}$, which is plotted as a yellow line in Fig. S16c. The SdH oscillations are harder to observe in $\sigma_{xx}^{Simu}$ due to the large background without the mobility edge. However, the situation is different in the simulation of magnetic susceptibility χ without the mobility edge. The measured χ of CaAs_3_ is $-1.1\times{10}^{-5}$ at 2 K, which is used as the background $\chi^{BG}$ in our simulation^15^. The dHvA oscillations at 0 K is simplified as a cosine function with the amplitude $\chi^{L}$, where $\chi^{L}=-\frac{1}{2}{(\frac{m_{e}}{m^{*}})}^{2}n\mu_{0}\mu_{B}^{2}/E_{F}^{0}$ is Landau diamagnetism susceptibility^16^, $m^{*}$ is effective mass, $n$ is carrier density and $E_{F}^{0}$ is Fermi energy. And $m_{e}$, $\mu_{0}$, $\mu_{B}$ is the mass of the free electron, the permeability of vacuum and Bohr magneton, respectively. If there is no mobility edge, all of the electrons can contribute coherent dHvA oscillations with amplitude $\chi^{L}=-6.76\times{10}^{-7}$ by consider $m^{*}=0.2 m_{e}$ and $n=n_{FS}$. The simulated susceptibility $\chi^{Simu}={\chi^{BG}+\chi}^{L}*\cos\left( 2\pi\frac{F}{B} \right)$ is plotted as a blue line in Fig. S16c and shows clear dHvA oscillations. Without the mobility edge, the dHvA oscillations are indeed more sensitive to Landau levels than SdH oscillations as observed in a traditional system.

**References**

1. Yuan, X. *et al.* Direct Observation of Landau Level Resonance and Mass Generation in Dirac Semimetal Cd_3_As_2_ Thin Films. *Nano Lett.* **17**, 2211–2219 (2017).

2. Witowski, A. M. *et al.* Quasiclassical cyclotron resonance of Dirac fermions in highly doped graphene. *Phys. Rev. B* **82**, 165305 (2010).

3. Xu, Q., Yu, R., Fang, Z., Dai, X. & Weng, H. Topological nodal line semimetals in the CaP3 family of materials. *Phys. Rev. B* **95**, 045136 (2017).

4. Quan, Y., Yin, Z. P. & Pickett, W. E. Single Nodal Loop of Accidental Degeneracies in Minimal Symmetry: Triclinic CaAs3. *Phys. Rev. Lett.* **118**, 176402 (2017).

5. Qu, D. X., Hor, Y. S., Xiong, J., Cava, R. J. & Ong, N. P. Quantum oscillations and hall anomaly of surface states in the topological insulator Bi2Te3. *Science* **329**, 821–4 (2010).

6. Analytis, J. G. *et al.* Two-dimensional surface state in the quantum limit of a topological insulator. *Nature Physics* **6**, 960–964 (2010).

7. Zhao, Y. *et al.* Anisotropic Fermi Surface and Quantum Limit Transport in High Mobility Three-Dimensional Dirac Semimetal Cd3As2. *Physical Review X* **5**, 031037 (2015).

8. Pezzini, S. *et al.* Unconventional mass enhancement around the Dirac nodal loop in ZrSiS. *Nat. Phys.* **14**, 178–183 (2018).

9. Hosen, M. M. *et al.* Experimental observation of drumhead surface states in SrAs3. *Sci Rep* **10**, 2776 (2020).

10. Tan, B. S. *et al.* Unconventional Fermi surface in an insulating state. *Science* **349**, 287–290 (2015).

11. Xiang, Z. *et al.* Quantum oscillations of electrical resistivity in an insulator. *Science* **362**, 65–69 (2018).

12. Sato, Y. *et al.* Unconventional thermal metallic state of charge-neutral fermions in an insulator. *Nat. Phys.* **15**, 954–959 (2019).

13. Jia, Y. *et al.* Evidence for a monolayer excitonic insulator. *Nat. Phys.* **18**, 87–93 (2022).

14. Sun, B. *et al.* Evidence for equilibrium exciton condensation in monolayer WTe2. *Nat. Phys.* **18**, 94–99 (2022).

15. Hosen, M. M. *et al.* Experimental observation of drumhead surface states in SrAs3. *Sci Rep* **10**, 2776 (2020).

16. Ashcroft, N. W. & Mermin, N. D. *Solid state physics*. (Saunders college publ, 1976).

**Fig. S1 | Crystal characterization. a**, X-ray diffraction patterns of the as-grown CaAs_3_ crystal, showing the (010) crystalline surface. **b**, Temperature dependence of resistivity down to 50mK in Sample C2, showing no sign of metallic behavior. **c**, Heat capacity *C_p_* from 10-150 K. **d**, The magnetic field dependence of conductivity corresponded to Fig. 2a. **e**, Hall curves of sample C1 at different temperatures from 0.3 K to 4.8 K. **f**, EDS spectrum of sample C2. The insets are SEM image of scanning region and corresponding images of chemical element mapping.

**Fig. S2 | Additional ARPES data.** **a-b**, Band dispersion at 45 K (**a**) and 70 K (**b**) measured by ARPES with the photon energy of 98 eV. **c**, Fermi surface contour.

**Fig. S3 | The activation fitting of Hall resistivity at 250~360 K (a) and 20~60 K in sample C1, respectively.**

**Fig. S4 | Calculation details of quantum oscillations.** **a**, The second derivative of MR in the range of 8~22 T. **b**, The Landau fan diagram by zero and extreme points in the second derivative curve, corresponding to an oscillation frequency of 23.4 T. **c**, The original MR curve (black solid line) and the background (red dash line) used to extract oscillating component. The green circles represent the zero point of the second derivative and the blue triangles represent the crossing point of MR at different temperatures. The MR background was derived by the Akima spline interpolation with five points, which include four zero points of the second derivative and a cross point of MR at different temperatures. **d**, The fitting of the Dingle damping factor with the Dingle temperature of $T_{D}$=3.9 K. The change of effective mass value for each oscillation peak was taken into account during the calculation.

**Fig. S5 | Effective mass fitting based on SdH oscillations normalized by** ***ρ_0_*.** **a**, SdH oscillations normalized by *ρ_0_*. **b**, The corresponding temperature factor fitting curves. **c**, Comparison of fitted effective mass between normalized by $\rho_{0}$ and $\rho_{BKG}$.

**Fig. S6 | Angle-dependent SdH oscillations of sample C3. a-b**, The magnetic field dependence of $\rho_{xx}$ with different angle *θ* and *φ* at 1.7 K and 0.3 K, respectively. The electric current is applied in the *a-c* plane. We used X-ray diffraction to determine the crystal orientation. The angle between the current and the *a-*axis is determined to be around 60°. **c-d,** corresponding second derivative of a-b shows clear SdH oscillations up to 90°.

**Fig. S7 | Comparison between band structure reproduced by fitted band parameters and the ARPES result. a**, Band structure calculated from $E_{\pm}\left( k \right)=E_{0}\pm\sqrt{{(E_{g}/2)}^{2}+\left( \hbar v_{F}k \right)^{2}}$ with fitted parameters. **b**, ARPES result in 10 K.

| $E_{g}(\mathrm{meV})$ | $v_{F}(\times{10}^{5}m/s)$ | $v_{F1}(\times{10}^{5}m/s)$ | $m_{D}$ |
| --- | --- | --- | --- |
| 165.5 | $2.69$ | 2.28 | 0.20$m_{e}$ |

**Table. S1 | Fitting result of band parameters from the magneto-infrared spectrum**. Here $v_{F1}$ is extracted from the fitting of $T_{0}$, and Dirac mass is obtained from $m_{D}=E{}_{g}/2v_{F}^{2}$ with $m_{e}$ is the bare mass of an electron.

**Fig. S8 | Density functional theory calculation. a-b**, Calculated band structure (**a**) and density of state (**b**), showing a DOS peak near the Lifshitz transition at the saddle point.

**Fig. S9 | Transport results of other samples.** **a**, Sign reversal of *R_H_* at 210K. The inset is the enlarged view. **b**, SdH oscillations with a frequency of 26.5 T. **c**, Longitudinal resistivity plotted as a function of temperatures. **d**, The Arrhenius plot of longitudinal resistivity.

**Fig. S10 | Consistent SdH oscillations and variational resistivity in different samples. a**, Comparison of SdH oscillations between the first measurement and the measurement after 4 years of the same sample C2. **b**, The normalized MR curves of four samples. **c**, Extracted SdH oscillations with similar frequencies. The variation of Fermi surface is little from sample to sample. **d**, Temperature dependence of resistivity of five samples. Note that the data of C3 is at 11 T while the rest is at 0 T.

**Fig. S11 | The variable range hopping (VRH) fitting of resistivity at low temperatures. a**, The Mott VRH fitting based on the formula $\rho=\rho_{0}e^{{(\frac{T_{0}}{T})}^{\frac{1}{4}}}$. **b**, The Efros-Shklovskii VRH fitting based on the formula $\rho=\rho_{0}e^{{(\frac{T_{0}}{T})}^{\frac{1}{2}}}$. Strong deviations are found in both fittings at low temperatures due to the saturation of $\rho_{xx}$.

**Fig. S12 | Schematic diagram of** **spatial inhomogeneity**.

**Fig. S13 | Effective mass fitted by the second derivative method. a**, The second derivative of magnetoresistance (MR) shifts π phase with extracted oscillations. **b**, The quantities $B^{4}\frac{\partial^{2}\rho_{xx}}{\partial B^{2}}$ at different temperatures. **c**, Fitted effective mass at different field by the second derivative method. Note that it is a stacked view with blue dashed lines representing the offset values. The effective mass fitting parameter is shown near each curve. **d**, comparison of effective mass derived by different methods.

**Fig. S14 | Effective mass fitted by the integral method. a**, range of integration around the peak position *B_0_*. **b**, extracted oscillations with shadow area *S_ocs_*. **c**, Fitted effective mass at different fields by the integral method. Note that it is a stacked view with blue dashed lines representing the offset values. The effective mass fitting parameter is shown near each curve. **d-e**, comparison of effective mass derived by different methods.

**Fig. S15 | Schematic plots of the electronic structure and calculated Fermi surface of CaAs_3_.** **a** and **c** Sketch of the band dispersion (left) and DOS (right) in CaAs_3_ when the Fermi level is below and beyond the van Hove singularity. **b** and **d**, Calculated Fermi surface by DFT when the Fermi level is below and beyond the van Hove singularity. The Fermi surface is highlighted by orange dashed circles.

**Fig. S16 | Measured and simulated quantum oscillations. a**, Measured conductivity and torque in experiment **b**, The corresponding sketch of the Fermi sphere with the mobility edge. **c**, Simulated conductivity and magnetic susceptibility without the mobility edge based on transport properties of CaAs_3_. **d**, The corresponding sketch of the Fermi sphere without the mobility edge.
